# American Society of Dental Surgeons

**Published:** 1842-09

**Authors:** 


### Miscellaneous Notices.

### AMERICAN SOCIETY OF DENTAL SURGEONS.

*Third Annual Meeting.*—We expected that this meeting would have been well attended, but, when we took into consideration the remoteness of the place at which it was to be held, we did not expect to see as many of the members of the association in attendance as were present on the occasion. We were therefore agreeably disappointed; and although we missed some whom we should have been highly gratified to have seen, and shaken by the hand, we had the pleasure of meeting others, whose acquaintance it gave us no small pleasure to make. The profession in Boston, or at least those of its members with whom we had the happiness to become acquainted, extended to the members of the society in attendance from a distance, the courtesies for which their city is so justly renowned. We shall ever cherish the remembrance of the friendly attentions received from them while there, and hope to have an opportunity at the next meeting of welcoming them, together with many of our other brethren, to the monumental city, at which place it is to be held.

The third annual meeting commenced its sittings at the Tremont House, Tuesday, the 19th of July, at 10 o'clock, A. M. The Minutes of the preceding meeting were read and approved. A committee was then, on motion of Dr. Hayden, appointed to arrange the business of the meeting, after which the Society adjourned to meet at 12 o'clock, M. at the principal lecture room of the Medical College in Mason-st. which had been politely tendered for the purpose, through the agency of Dr. G. V. C. Smith, editor of the "Boston Medical and Surgical Journal."

Among the members in attendance, we call to mind the names of the following—H. H. Hayden, M. D, of Baltimore, President; Eleazar Parmly, M. D, of New York, 1st Vice-Pres't; Lewis Roper, M. D, of Philadelphia, 2nd Vice-President; Solyman Brown, M. D., Recording Secretary; Edward Maynard, M. D., of Washington City; Jas. S. Gunnell, M. D., of Washington City; Leonard Mackall, M. D., of Baltimore; N. C. Keep, M. D. of Boston; Jahial Parmly, D. D. S., of New York; Elisha Baker, M. D., of New York; T. B. Hamlin, of Wythville, Va.; James Alcock, of New York; J. N. Frink, of Portland, Me.; Chas. Rowall, of New York; Dr. Peabody, of Boston; Dr. J. E. Fisk, of Salem, Mass.; E. G. Tucker, M. D., of Boston; Dr. John Lovejoy, of New York; H. N. Fenn, M. D., of Rochester, N. Y.; J. M. Peak, of Cooperstown, N. Y.; M. K. Bridges, of Brooklyn, N. Y.; J. W. Smith, of Amherst, Mass.; Joshua Tucker, M. D., of Boston; A. Wescott, M. D., of Syracuse, N. Y.; A. C. Hawes, of Providence, R. I.; A. Hill, Norwalk, Conn.; O. P. Laird, M. D., Columbus, Ga.; Wm. Pitt Greenwood, of Boston; Benjamin Lord, of New York; C. W. Grant, M. D., of Newburg, New York; Elisha Blake, M. D., of New York; E. Noyes, M. D. of Baltimore; and G. G. Brewster, M. D. of Portsmouth, N. H.

There may have been others present on the occasion, but if there were, their names have escaped our recollection.

Letters of apology for non-attendance were received from Dr. S. P. Hullihen, of Wheeling, Va.; Dr. N. A. Fisher, of Providence, R. I.; B. B. Brown, M. D., of St. Louis, Mo.; Benj. Strickland, of Cleveland, Ohio; E. J. Dunning, M. D., of Montrose, Pa.; Edward Taylor, M. D., of Natchez, Miss.; John Harris, M. D., of Georgetown, Ky.; S. Dillingham, of Philadelphia; S. M. Shepherd, of Petersburg, Va.; and Dr. James D. McCabe, of Richmond, Va.

Agreeably to adjournment, the Society met at 12 o'clock in the Mason street Medical College, whereupon, the meeting having been called to order by the President, the names of the members were read by Dr. Brown, the Recording Secretary, and there being a quorum present, the report of the committee of arrangement was called for. The substance of this was, that the Introductory Address by Dr. Eleazar Parmly, be delivered on Wednesday, at 11 o'clock, A. M.; that a dissertation proposed to be read by Dr. Hayden, be delivered at 12 o'clock, M. and another by Dr. Harris, on the Diseases of the Maxillary Sinus, at 4 o'clock, P. M.; and an Essay by Dr. Maynard, on Salivary Calculus, at 5 P. M.; same day. The hour of 11 A. M. Thursday, was assigned Dr. Brown, for the reading of an Essay on the surest methods of degrading the profession of dental surgery.

The report of this committee having been adopted, Mr. B. Lord, of New York, was reported to the Society by the Executive Committee, as a suitable candidate for membership.

At two o'clock, the Society adjourned till half past three, same day, to meet at the same place.

*Afternoon Session.*—At half past three o'clock, the society agreeably to adjournment, assembled. The Chair having been taken by Dr. Eleazar Parmly, Dr. L. Mackall, of Baltimore, announced to the society the demise of A. B. Hayden, of Savannah, Ga. late a member of the Association, who since its last annual meeting had departed to the world of spirits. Dr. M. concluded by offering a series of appropriate resolutions, which were unanimously adopted by the Society.

Dr. Maynard, of Washington City, was the only member of the Executive Committee in attendance; consequently the whole of the business of that important committee devolved upon him, but it could not have fallen into better hands. But for the prompt and systematic manner in which the business of this committee was presented to the Society, its meeting would have been continued, at least, a day longer than it was.

After the adoption of the resolutions offered by Dr. Mackall, the following gentlemen, after having been favourably reported by the aforesaid executive committee, were successively put in nomination and each duly elected, viz. Wm. G. Lord, of Newark, N. J.; J. W. Smith, of Amherst, Mass.; J. M. Peak, of Cooperstown, N. Y.; W. E. Ide, of Zanesville, Ohio; W. H. Goddard, of Louisville, Ky.; David Marrow, M. D., of Tuscaloosa, Ala.; Dr. Foster, of Utica, N. Y.; Dr. Blakesly, of Utica, N. Y.; Dr. G. E. Hawes, of New York; Chas. C. Allen, of New York; P. N. Williams, of St. Johns, Porto Rico; W. J. B. Lord, of New York; Amos Wescott, M. D., of Syracuse, N. Y.; A. Hill, of Norwalk, Conn.; E. G. Kelly, M. D., of Newburyport, Mass.; C. Merritt, of Bridgeport, Conn.; William H. Lintot, of London, was then elected an honorary member, as was also, Mr. David Rosseter, of New York.

The society now adjourned to the Tremont House, to meet at 8 o'clock, P. M.

*Evening Session.*—The society having met agreeably to adjournment, the following gentlemen were duly elected, viz. George W. Smith, of New Orleans; Mr. Lloyd, of Manchester, England; James Atkinson, of Leeds, Eng.; John G. Wayt, of Richmond, Va.; and John McConnell, of Richmond, Va.

The corresponding secretary then read letters from Alexander Nasmyth, and Leonard Koecker, M. D., of London; C. Bromly, of Southhampton, Eng; and from various other gentlemen, both from abroad and in the United States.

At half past ten o'clock the society adjourned to meet the following morning at 8½ o'clock, at the Medical College, Mason street.

*Wednesday Morning Session, July 20th.*—At half past eight o'clock, the society, agreeably to adjournment, met in the Mason street Medical College, where the following gentlemen were proposed by the executive committee for membership. Wm. O. Laird, of Athens, Ga.; C. T. Cushman, of Columbus, Ga.; J. C. Ross, of Frankfort, Ky. Satisfactory evidence of their qualifications having been adduced, they were duly elected members of the society. After which George Waite, Esq. of London, was chosen an honorary member.

A resolution was now offered by Dr. Jahial Parmly, of New York, which was unanimously passed, to the effect that no letters, pertaining to the society or American Journal and Library of Dental Science, except they contain money, be taken from the post office by the Editors of the Journal, or any of the officers of the society, unless the postage has been paid by the writer, after the publication of the next number of the Journal.

This resolution is not of course intended to apply to letters coming from abroad, for in such cases, though the writer pay the postage to this country, a charge is here made that he cannot by any possibility pay.

The President now presented sundry papers relating to alleged violations of professional etiquette on the part of one of the members of the society, which were referred to the Committee on Grievances.

The hour of eleven having arrived, Doctor Eleazar Parmly delivered an Introductory Address—for which see first article in this number of the Journal. Dr. Parmly having concluded his address, Dr. Harris moved that it be published with the transactions of the society, accompanied by a portrait of the author, which resolution was unanimously passed.

Dr. Hayden, then read a lecture on the Diseases of the Antrum Maxillare, which having been concluded, the society adjourned till 3½ o'clock, P. M.

*Afternoon Session.*—The society met agreeably to adjournment, and on motion of Dr. Brown, Simon Van Namee, of Rhinebeck, was duly elected a member of the society.

The hour of four having arrived, Dr. Harris commenced the reading of a paper on the Diseases of the Maxillary Sinus, but after having proceeded one hour, begged to be excused from its further reading, which request having been granted, it was moved that the whole dissertation be published, with the transactions of the society. This resolution having been adopted, Dr. Maynard, of Washington City, read a highly interesting Essay on Salivary Calculus, which was ordered by the society to be published as a tract, for distribution by the members of the society.

The society now adjourned to meet by special invitation, at the house of Dr. N. C. Keep, No. 1 Tremont street, at 6½ o'clock, P. M.

*Evening Session.*—The society met agreeably to adjournment, at the house of Dr. Keep. The first business which here engaged its attention was the appointment of a committee to audit the accounts of the Treasurer, the two Secretaries, and Editors of the American Journal and Library of Dental Science. Drs. Jahial Parmly, Leonard Mackall, and L. Roper, were appointed upon that committee.

Dr. C. A. Harris was then appointed by the society to deliver the opening address at the next annual meeting, and the following gentlemen to deliver dissertations on the occasion: namely, N. C. Keep, M. D., of Boston; John C. McCabe, of Richmond, Va.; Elisha Baker, M. D., of New York; O. P. Laird, M. D., of Columbus, Ga.; and Amos Wescott, M. D., of Syracuse, N. Y.

On motion of Dr. Maynard, Resolved, that Dr. Bond, of the Baltimore College of Dental Surgery, be invited to deliver a dissertation on the Morbid Sympathies between the Dental Apparatus and other parts of the body. Drs. Mackall and Noyes were appointed by the society to communicate the request contained in this resolution to Dr. Bond.

Dr. E. Baker, of New York, was next appointed to prepare an Essay on the Diseases of the Gums.

An essay on the best method of remedying a protrusion of the lower jaw, by Dr. Jas. S. Gunnell, was then read and ordered to be published among the proceedings of the society.

On motion of Dr. Joshua Tucker, a resolution was offered and passed, to the effect, that an examining and recommending committee, composed of three members, be appointed, from each of the four cities of Baltimore, New York, Philadelphia and Boston; whose duty it shall be to report to the executive committee at each annual meeting of the society, the names of such persons, as may be desirous of becoming members, as they may deem worthy of membership, in the respective districts where said committees reside.

The following gentlemen were placed on said committees. Boston, Drs. J. Tucker, N. C. Keep, and J. E. Fisk; New York, Drs. E. Parmly, S. Brown, and E. Baker; Philadelphia, Drs. L. Roper, H. S. Burr, and M. Dillingham; Baltimore, Drs. C. A. Harris, L. Mackall, and E. Noyes.

The society now proceeded to the election of its officers for the ensuing year; whereupon, H. H. Hayden, M. D., was elected President; Eleazar Parmly, M. D., 1st Vice-president; L. Roper, M. D., 2nd Vice-president; N. C. Keep, M. D., 3rd Vice-president; C. A. Harris, M. D., Corresponding Secretary; S. Brown, M. D., Recording Secretary; L. Mackall, M. D., Treasurer; J. H. Foster, M. D., Librarian.

The society having completed the election of its officers, it adjourned until nine o'clock, A. M. Thursday, July 21st, to meet at the Mason street Medical College.

*Thursday Morning,* 9 *o'clock.*—The society met agreeably to adjournment. After the meeting was called to order, by the President, the following gentlemen were nominated and duly elected members of the society, viz. Wm. H. Dwinelle, of Cazenovia, N. Y.; Wm. H. Elliot, Plattsburg, N. Y.; Hiram B. Lathrop, Brooklyn, N. Y.; Dr. N. Dodge, New York; Wm. Johnson, Hagarstown, Md.; L. Murrill, Petersburg, Va.; H. H. Young, M. D., Troy, N. Y.

A motion was then made to the effect that the constitution be so altered as to give to the society a permanent locality and place of meeting, after the next meeting which the society had determined should be in Baltimore, and that said permanent place of meeting be either in the city of New York, or Philadelphia. The resolution having prevailed, the members proceeded to determine by ballot which of the two cities above mentioned should be the place of location, it was determined in favour of New York, by a majority of eight votes.

Dr. Baker then moved that hereafter there be three editors to the Journal, instead of two, which resolution having been assented to by the society, an election was gone into for the same; and, to the two former editors, Leonard Mackall, M. D., was added.

A letter was next read from the Boston Society of Natural History, inviting the members of the American Society of Dental Surgeons, then present in Boston, to visit the collection of curiosities belonging to the first named society. Whereupon it was resolved that the invitation be accepted; the thanks of the society was then returned to the Boston Society of Natural History for their polite invitation.

The executive, examining and publishing committees of the past year were continued for the year ensuing.

At twelve o'clock, M. Dr. Brown read a dissertation on the surest methods of degrading the dental profession. The reading of this having been concluded, Dr. Maynard, moved that it be printed with the transactions of the society, in the American Journal of Dental Science, and that it also be published in pamphlet form, and that the members be requested to subscribe for it for distribution. Whereupon, three thousand one hundred and eighty copies were subscribed for by the members present.

Among the instruments exhibited to the society, was a drill stock, invented by Dr. Maynard. This received the approval of the society, and was pronounced by the members present to be the most complete instrument for the purpose intended, known to the society.

Dr. N. C. Keep was appointed to present the thanks of the society to the Faculty of the Mason street Medical College, for the use of their Lecture Room.

The business of the association having been completed, the society adjourned to meet in the city of Baltimore, on the third Tuesday of July, 1843.

In the account which we have given of the proceedings of the society, we have only noticed such parts as we thought would be likely to be interesting to the readers of the Journal.

